# Radiotherapy of MRI-detected involved internal mammary lymph nodes in breast cancer

**DOI:** 10.1186/s13014-017-0934-5

**Published:** 2017-12-19

**Authors:** Sean Sachdev, Chelain R. Goodman, Erin Neuschler, Kapila Kalakota, Daniel Cutright, Eric D. Donnelly, John P. Hayes, Adam E. Prescott, Gianna Mirabelli, Jonathan B. Strauss

**Affiliations:** 1 Department of Radiation Oncology, Northwestern University Robert H. Lurie Comprehensive Cancer Center, 251 E. Huron Street LC-178, Chicago, IL 60611 USA; 2 Division of Breast Imaging, Department of Radiology, Northwestern University Feinberg School of Medicine, 676 N. St Clair Street #800, Chicago, IL 60611 USA; 3 Radiation Oncology Consultants Ltd., 120 N. Oak Street, Hinsdale, IL 60521 USA

**Keywords:** Breast, Breast neoplasms, Lymph nodes, Magnetic resonance imaging, Radiotherapy

## Abstract

**Background:**

The internal mammary (IM) lymph node chain, along with the axillary nodal basin, is a first-echelon breast lymphatic draining site. A growing body of evidence supports irradiation of this region in node-positive breast cancer. This study evaluated the effectiveness of radiotherapy in treating magnetic resonance imaging (MRI)-detected abnormal IM lymph nodes in newly-diagnosed non-metastatic breast cancer.

**Methods:**

A structured query was performed on an electronic institutional database to identify women with radiographic evidence of abnormal IM node(s) on breast MRI from 2005 to 2013. Manual review narrowed inclusion to patients with a primary diagnosis of non-metastatic breast cancer with abnormal IM node(s) based on pathologic size criteria and/or abnormal enhancement.

**Results:**

Of the 7070 women who underwent pre-treatment MRI, 19 (0.3%) were identified on imaging to have a total of 25 abnormal pre-treatment IM lymph nodes, of which 96% were located in the first two intercostal spaces and 4% in the third space. A majority of the primary tumors were high-grade (94.7%) and hormone-receptor negative (73.7%), while 47.4% overexpressed HER-2/neu receptor. Axillary nodal disease was present in 89.5% of patients, while one patient had supraclavicular involvement. At a median follow-up of 38 months, 31.6% of patients had developed metastatic disease and 21.1% had died from their disease. Of the patients who received IM coverage, none had progressive disease within the IM lymph node chain.

**Conclusions:**

Radiologic evidence of pre-treatment abnormal IM chain lymph nodes was associated with advanced stage, high grade, and negative estrogen receptor status. The majority of positive lymph nodes were located within the first two intercostal spaces, while none were below the third. Radiation of the IM chain in combination with modern systemic therapy was effective in achieving locoregional control without surgical resection in this cohort of patients.

## Background

The internal mammary (IM) lymph node chain, along with the axillary nodal basin, is a primary breast lymphatic draining site. [1] IM nodal dissection was routinely pursued in the 1950s due to reports that as many as 33% of patients had IM nodal involvement on survey biopsies. [2] Multinational trials subsequently revealed that extended radical (Urban) mastectomy with IM nodal dissection did not improve survival compared to radical (Halstead) mastectomy [3–6], eventually leading to its disuse.

Historically there has been significant controversy regarding when to electively treat the IM nodal chain with radiotherapy. This controversy stems both from uncertainty concerning the therapeutic value of treating the IM chain as well as concern over the incremental additional dose delivered to heart and lung. [7] Recent advances in the delivery of radiotherapy, such as deep inspiration-breath hold, have abrogated the concerns about cardiac dose, although the magnitude of the benefit remains unclear. Prospective randomized data have demonstrated that nodal radiotherapy improves locoregional control as well as distant metastasis-free survival and reduces breast cancer mortality. [8, 9] Furthermore, the large absolute overall survival advantage of post-mastectomy radiotherapy in node positive women suggests that the inclusion of the regional nodal beds may provide at least part of this observed benefit. [10–13] These reports, however, were based on outcomes of nodal treatment in aggregate; limited data exist regarding the incremental benefit associated with including the IM chain. The Danish Breast Cancer Cooperative Group (DBCG)-IMN study, which prospectively assigned patients with left-sided breast cancer to receive radiation to the IM chain, demonstrated a significant survival benefit of IM chain irradiation. [14] To date, however, there are no randomized clinical trials evaluating this question, and there remains a lack of consensus as to which patients may benefit from the elective radiation of this region. [15–17]

In this study, radiotherapeutic treatment of the IM chain was evaluated in a related setting. Rarely, patients are found to have abnormal pre-treatment IM lymph nodes on imaging. From a large pool of patients who underwent pre-treatment MRI evaluation, patients who were found to have radiologic evidence of internal mammary node involvement were identified. Clinicopathologic data, radiotherapeutic treatment parameters, and clinical outcomes were analyzed to help clarify the effectiveness of radiotherapy and modern systemic therapy in treating involved IM nodes.

## Methods

### Patient selection and search query

Patients were retrospectively identified from an electronic database with records of 7070 women who underwent pre-operative breast MRI from 2005 to 2013 at a single institution. All data collection and analyses were performed after review by and approval of the institutional review board (IRB) and in accordance with medical research principles outlined by the Declaration of Helsinki. [18] Numerous search strings, such as “internal mammary node” and “IM node,” were used to identify studies with abnormal internal mammary lymph node(s) as documented by a breast radiologist. A structured query was used to obtain a comprehensive list from which duplicates were excluded.

### Imaging, dosimetry, and clinical review

MR studies were retrieved and manual review narrowed case inclusion to women with a primary diagnosis of non-metastatic breast cancer with abnormal IM node(s) based on size and/or abnormal contrast enhancement as seen on axial fat suppressed T1-weighted and STIR Axial images. Each MRI was individually reviewed with an experienced breast radiologist and a final determination was reached regarding radiographic diagnosis of IM chain involvement. For each patient, the number and size of the involved IM lymph node(s), intercostal space location(s), radiologic evidence of extracapsular extension (ECE), as well as quadrant(s) involved by the primary malignancy were noted. Characteristics of the primary tumor including grade, evidence of lymphovascular invasion (LVI), and hormone receptor profile were recorded. Treatment-related variables including the type of breast surgery and axillary node evaluation as well as the type and sequence of systemic therapy.

All available radiation treatment planning data were de-archived. Dosimetric parameters including prescribed dose, mean dose received, and dose prescribed to 95% of the treated volume (D95) were recorded for the breast/chest wall clinical target volume (CTV), axillary and supraclavicular (SCV) nodal chain CTV, and IM nodal chain CTV. The use of separate fields or boosts to cover involved lymph nodes was also documented. Clinical and pathologic stages were assigned per the American Joint Committee on Cancer (AJCC) 7th edition staging criteria. Follow-up date was defined as the last documented note in our institution’s electronic medical record system or date of death. Follow-up time was defined as the number of months between date of diagnosis and last follow-up date. Date and location of recurrence, metastasis, or death was documented. Survival curves were plotted as unadjusted Kaplan-Meier estimates.

## Results

Nineteen women with a median age of 52 years (range = 33–88) were identified to have abnormal pre-treatment IM lymph nodes by database screen and subsequent manual review (Table 1). All patients had a clinical tumor classification of T2–4. The pathologic stage ranged from IB to IIIC with the exception of 5 women (26.3%) who demonstrated complete response after neoadjuvant treatment. The median size of the greatest dimension of the primary tumor as measured clinically was 6.7 cm (range = 2.10–13.30 cm). High histologic grade was assigned to 94.7% (18/19) of primary tumors, and 52.6% (10/19) demonstrated LVI. ECE of axillary nodal disease was present in 31.6% (6/19) of patients. Fifteen patients (73.7%) had hormone-receptor negative tumors, of whom (10/19) were HER-2/neu positive and 7/19 had triple negative disease. Seventeen patients (89.5%) had identifiable axillary nodal disease, while one patient had both axillary and supraclavicular nodal involvement.

**Table 1:** Patient characteristics

No.	Age	cTN	pTN	No. IM nodes	IM node size	No. Axillary nodes	Inner Quad	LVI	Radiation to IM node	Status
1	34	cT3N3	ypT0N1	1	1.80	1/35	Yes	Yes	Yes	
2	37	cT4N3	ypT0N1	1	1.40	1/24	Yes	No	Yes	
3	42	cT3N3	ypT3N1	1	2.40	1/3	No	Yes	Unk	Metastatic
4	52	cT3N3	ypT3N2	2	1.40, 0.90	8/8	No	Yes	Yes	
5	57	cT3N3	ypT0N3	1	1.40	23/28	Yes	Yes	Yes	Metastatic
6	61	cT2N2	ypT1N0	2	1.40, 1.30	1/1	No	Unk	Unk	
7	62	cT2N3	ypT1N0	1	1.00	0/13	No	Yes	Yes	
8	65	cT3N2	ypT3N1	2	1.70, 2.90	2/24	Yes	Yes	No	LRR to IM chain
9	66	cT2N3	ypT1N0	1	2.60	0/3	No	No	Yes	
10	34	cT2N3	ypT0N0	1	2.10	0/18	Yes	No	Yes	
11	44	cT3N2	ypT0N0	2	2.00, 0.60	0/1	Yes	Unk	Yes	
12	49	cT2N2	ypT0N0	2	1.00, 1.00	0/20	No	No	Partial	
13	50	cT3N2	ypT0N0	1	0.90	0/5	Yes	No	Yes	Metastatic
14	52	cT3N2	ypT0N0	1	0.60	0/15	No	Unk	Yes	
15	43	cT2N2	pT2N1	1	3.00	2/5	No	Yes	Yes	LRR to tumor bed
16	49	cT2N2	pT2N2	1	0.50	4/16	No	No	Unk	
17	52	cT3N3	pT2N2	1	0.40	8/22	No	Yes	Yes	
18	53	cT3N2	ypTxN2	2	0.70, 0.70	5/8	Yes	Yes	Unk	Metastatic
19	88	cT3N3	pT3N3	1	0.40	18/35	No	Yes	Yes	Metastatic

*No* Number, *cTN* Clinical Tumor and Nodal classification, *pTN* Pathologic tumor and nodal classification, *yp* Pathologic tumor and nodal classification following neoadjuvant chemotherapy, *Quad* Quadrant, *LVI* Lymphovascular invasion, *LRR* Locoregional recurrence; *Unk* Unknown

**Fig. 1:**
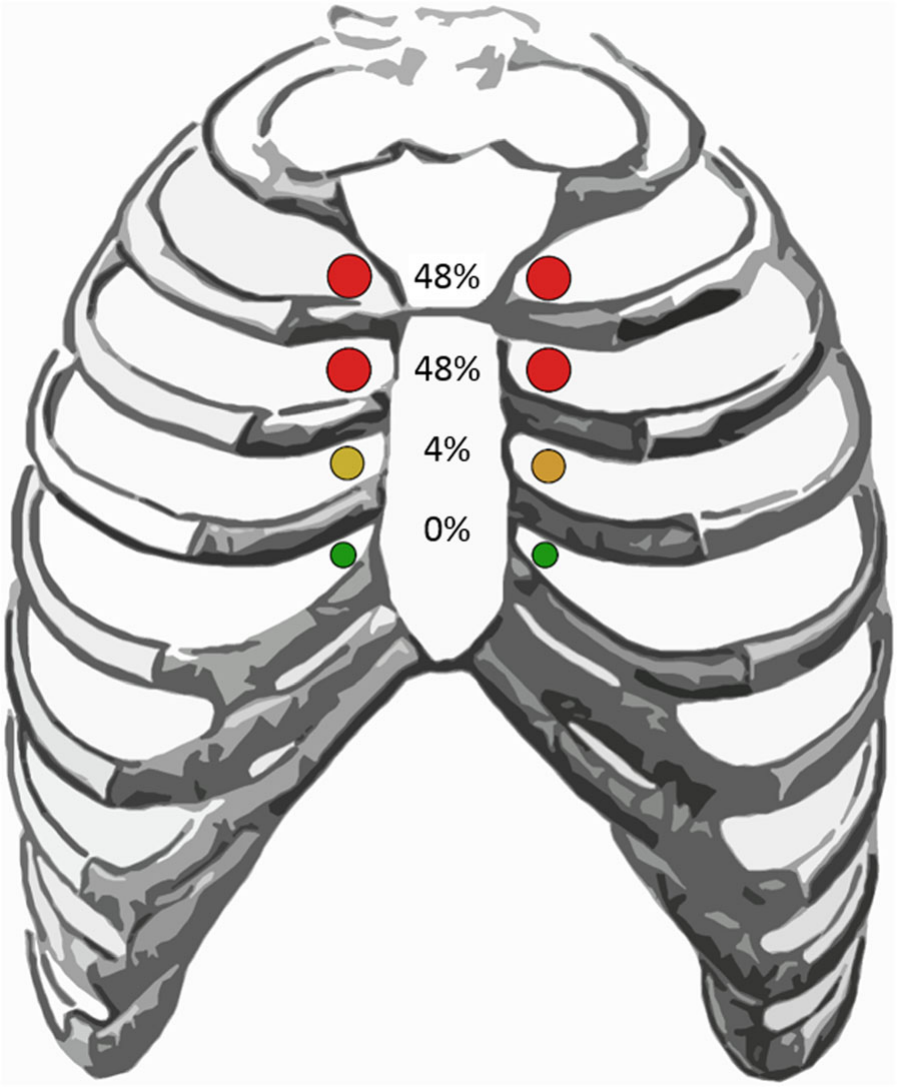
Intercostal space distribution of involved nodes. Schematic diagram of the intercostal space distribution of the 25 abnormal internal mammary lymph nodes identified on pre-treatment breast MRI

Twenty-five abnormal lymph nodes were radiographically identified in total. Twelve (48%), twelve (48%), and two (4%) nodes were located in the first three intercostal spaces, respectively (Fig. 1). No involvement was found below the 3rd intercostal space. All IM nodal involvement was ipsilateral to the known breast cancer. Eleven women (57.9%) had malignancies that did not cross into the inner quadrant of the involved breast. Twenty-three of 25 nodes identified (92.0%) were ≥0.5 cm in at least one dimension. The mean short and long axis lengths were 0.82 (standard deviation (STD) = 0.48 cm) and 1.36 cm (STD = 0.77 cm), respectively. The mean short axis to long axis ratio (S/L) was 0.63 (STD = 0.19). The mean volume was 1.184 cm^3^ (range = 0.02–5.53 cm^3^) as calculated by the ellipsoid formula.

All but one patient (94.7%) received chemotherapy; 78.9% (15/19) of patients received neoadjuvant chemotherapy and 15.8% (3/19) were treated with post-operative chemotherapy (Table 1). Of those patients treated with neoadjuvant chemotherapy, 67% (10/15) demonstrated a pathologic complete response. All but one patient with an estrogen receptor (ER)-positive tumor (3/4) received adjuvant anti-endocrine therapy, while all Her2-overexpressing patients received adjuvant trastuzumab.

**Fig. 2:**
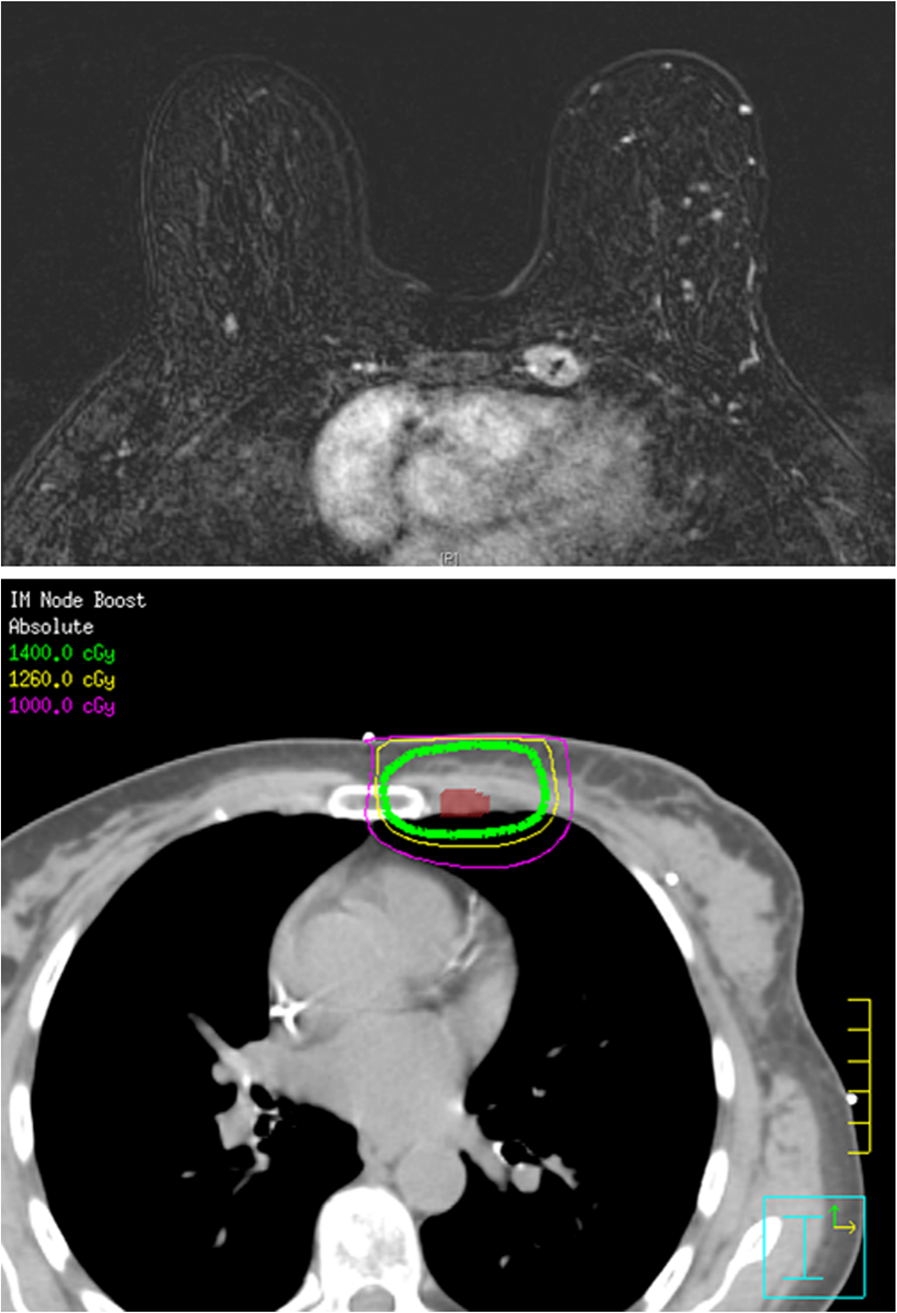
Representative MRI and treatment plan. Representative internal mammary node identified on MRI (Top) and treatment plan of dedicated 14Gy boost (Bottom). Maroon contour = IM node GTV

Eighteen patients received adjuvant radiation to the breast or chest wall with photons (6–10 Mega-volts (MV)) using tangent beams with or without matched electron fields (9–12 Megaelectron-volts (MeV)); one patient was treated with intensity modulated radiotherapy technique. Seventeen patients (89.4%) were treated to the axillary and supraclavicular lymph node basins with photons using oblique fields (6–18 MV). Boosts to the mastectomy scar or IM nodal chain were given using electrons (9–20 MeV). Of the 15 patients for whom dose prescriptions were available, the median dose to the breast or chest wall was 50.4 Gray (Gy;range = 45–50.4Gy) prior to a median 10Gy boost (range = 0-16Gy) to the surgical bed or mastectomy scar. The median dose to the axillary/supraclavicular basin was 50.4Gy (range = 45–50.4Gy). 73.6% of patients (14/19) received dedicated treatment of the IM nodal chain to a median dose of 50.4Gy (range = 45–50.4Gy), while coverage of the IM chain was unknown for 4 patients. A single patient was treated at an outside institution without coverage of the IM chain. Four of the 14 patients who were treated to the IM nodal chain (28.6%) were prescribed a boost to a median dose of 14.0Gy (range = 10-14Gy). The median total dose prescribed to the IM nodal chain was 50.4Gy (range = 45–64.4Gy). The median mean dose received to the IM nodal chain was 53.5Gy (range = 31.1–75.8Gy). The median D95 was 45.8Gy (range = 12.3–70.8Gy). A representative MRI and treatment plan of a dedicated 14Gy boost to an involved IM node is shown in Fig. 2.

After a median follow-up of 38 months (Interquartile range = 30.0–53.3), 18 of 19 (94.7%) patients maintained local control of the IM nodal chain and 17 of 19 (89.4%) were free from locoregional recurrence. No patient that received dedicated treatment of the IM chain experienced a recurrence or progression at the site of the involved node(s). One patient who did not receive coverage of the IM chain developed progressive disease within the IM chain as well as at distant sites, and died 28 months following diagnosis. One patient experienced a local recurrence at the lumpectomy site 28 months following her original diagnosis. Of all 19 patients, 6 (31.6%) developed metastatic progression to the brain, lung, and/or bone, while 4 (21.1%) died from their disease by the time of last follow-up (Table 2). Of the 14 patients who received dedicated radiation to the IM chain, 13 (96.9%) achieved locoregional control, while 3 (21.4%) developed metastatic disease, and 2 (14.3%) died (Table 2). Since median survival was not observed, restricted mean survival times were estimated using the Kaplan-Meier method. Restricted mean overall survival was 76.7 months (95% Confidence Interval (CI) = 61.0–92.4), restricted mean locoregional recurrence free survival was 86.0 months (95% CI = 74.2–97.8), and restricted mean disease free survival was 65.2 months (95% CI = 47.8–82.6).

**Table 2:** Patient outcomes

Event	All patients (*N* = 19)	IM radiation (*N* = 14)	No IM radiation (N = 1)	Unknown IM radiation (*N* = 4)
IM chain recurrence	1 (5.3%)^a^	0 (0%)	1 (100%)	0 (0%)
Locoregional recurrence	2 (10.5%)	1 (7.1%)	1 (100%)	0 (0%)
Distant recurrence	6 (31.6%)	3 (21.4%)	1 (100%)	2 (50.0%)
Death	4 (21.1%)	2 (14.3%)	1 (100%)	1 (25.0%)

*IM* Internal mammary. ^a^Did not receive radiation to IM chain

## Discussion

The inclusion of the IM nodal chain as part of regional nodal irradiation for patients with breast cancer has been historically controversial in part due to the difficulty in detecting IM nodal involvement. As breast MRIs are increasingly being performed prior to treatment, radiologic identification of involved IM lymph nodes is providing a new window into the phenomenon of IM nodal involvement. [19] In this single institution analysis of 7070 women who underwent pre-treatment MRI, 19 patients were identified by search query and manual review as having clinical involvement of the IM lymph node chain. The cases identified tended to skew towards a more clinically advanced and/or aggressive tumor phenotype. All patients had clinical T2-T4 primary tumors and all but 2 had known axillary or supraclavicular disease. These cases also skewed toward more aggressive subtypes as 94.7% were grade 3, 73.7% were hormone receptor-negative, and 52.6% demonstrated overexpression of the Her-2/Neu receptor. The vast majority (96%) of the involved IM nodes were located in the first 2 intercostal spaces. All but one patient received chemotherapy, and all Her-2/Neu positive patients received Herceptin. Of the patients who received dedicated radiation to the IM chain, none experienced a recurrence or progression at the site of clinical nodal disease. The sole patient with progression of IM nodal disease had not received radiotherapy to the IM nodal chain. One patient experienced recurrence at the lumpectomy bed. Systemic control, however, remained problematic, as six patients (31.6%) developed distant disease and four (21.1%) died within 1–3 years of diagnosis.

In a large surgical (pathologic) series of women who had undergone an extended mastectomy, involvement of the IM region portended significantly worse outcomes: disease-free-survival was 23% at 20 years compared to 76% in IM-negative patients. [20] Our results corroborate the finding that IM nodal involvement portends a worse prognosis, although improved staging and systemic therapy, among other advances, account for far superior outcomes in this series as compared to historical data. There are relatively few reports of IM nodal involvement identified on imaging, however, our data appear to be congruous with those of other institutions. Lee et al. demonstrated that malignant IM lymph nodes identified on MRI had a short/long (S/L) axis ratio that was significantly greater than the ratio for benign IM nodes (0.45 ± 0.10 vs 0.59 ± 0.17). [21] The mean S/L ratio of the 26 nodes identified in our series (0.63 ± 0.19) is consistent with this report. Bellon et al. reported on outcomes of seven women with locally advanced breast cancer and increased uptake in the IM nodes seen on positron emission tomography. [22] Follow-up data were available on 6 of these seven women; all three who received no IM nodal radiotherapy experienced IM nodal failure. In conjunction with our series, this point highlights the need to include involved IM nodes in the radiotherapy field. There was an association between large tumor size and IM nodal involvement, a finding that mirrors the more advanced tumor classification seen in our series. Zhang et al. reported on 112 women with involved IM nodes as seen on a variety of imaging studies. [23] In that series, the vast majority of patients (87%) had T2-T4 tumors, almost all (91%) had involved axillary and/or SCV nodes, 75% were grade 3, and 56% were ER-negative. Five-year IM nodal control in this group, most of whom received IM nodal radiotherapy, was 84%. These patient and tumor variables are similar to those seen in our study. The high rate of IM nodal control highlights the effectiveness of the combination of targeted radiotherapy and modern systemic therapy.

The results of the present study, in combination with other reports, have interesting implications. First, they validate the current understanding that the upper 3 intercostal spaces of the ipsilateral IM nodal chain account for the vast majority of involved IM nodes and most of those lie in the upper 2 spaces. Thus, if elective IM nodal radiotherapy is to be used, then inclusion of the upper 3 intercostal spaces may be sufficient. Intriguingly, our series identified a preponderance of ER-negative tumors in the patients presenting with IM nodal involvement. This mirrors a planned subset analysis of the MA-20 trial, which found a sizable survival advantage for the addition of nodal radiotherapy that was limited to ER-negative patients. [8] Although patients within the MA-20 trial did not have clinically involved IM nodes, MRI staging was not performed and nodes may have been clinically occult but radiologically suspicious. If validated in other studies, this finding could support the use of elective nodal radiotherapy in these patients. As in our series, Zhang et al. found a high rate of regional control with radiotherapy. This is attributable to the remarkable synergy of accurate modern radiotherapy with 3-dimensional (3D) planning in combination with increasingly effective and targeted systemic therapy. Given the efficacy of these treatment modalities and the high morbidity associated with surgical excision of the IM nodal chain, definitive irradiation of involved IM nodes, when identified, may therefore be the optimal choice of treatment compared to surgery. [24]

There are several limitations to the present study. As a retrospective review, there is an inherent selection bias regarding case identification and are limited by the paucity of identifiable patients from a single institution. Our sample size was further limited as very few women receive MR imaging at our institution as part of the work-up for a breast malignancy; indeed, some of the patients included in our cohort received an MR for screening purposes. The overall rate of IM nodal involvement identified in this series is therefore not generalizable to a population with known breast cancer.

## Conclusions

In summary, in this series a majority of patients with involved IM nodes as identified on pre-treatment MRI had advanced primary tumors that were of aggressive grade and tumor subtype. Involved nodes were limited to the upper three intercostal spaces with the majority found within the first two spaces. IM nodal chain control was achieved for all patients for whom the IM chain was irradiated. A relatively high risk of distant metastatic spread, however, persisted in this population. The combination of modern systemic therapy with radiation including the IM nodes may therefore be sufficient for achieving locoregional control in patients with either clinical evidence of or occult involvement of the IM chain.

## Abbreviations

3D: 3-dimensional
AJCC: American Joint Committee on Cancer
CI: Confidence Interval
CTV: Clinical target volume
DBCG: Danish Breast Cancer Cooperative Group
ECE: Extracapsular extension
ER: Estrogen receptor
Gy: Gray
IM: Internal Mammary
IRB: Institutional Review Board
LVI: lymphovascular invasion
MeV: Megaelectron-volt
MRI: Magnetic Resonance Imaging
MV: Mega-volt
SCV: Supraclavicular
